# Pharmacokinetics, Tissue Distribution, Metabolism and Excretion of a Novel COX-2 Inhibitor, Vitacoxib, in Rats

**DOI:** 10.3389/fvets.2022.884357

**Published:** 2022-04-08

**Authors:** Jianzhong Wang, Jingyuan Kong, Yuxin Yang, Yu Liu, Jicheng Qiu, Xiaohui Gong, Lu Zhang, Jing Li, Feifei Sun, Xingyuan Cao

**Affiliations:** ^1^Shanxi Key Laboratory for Modernization of TCVM, College of Veterinary Medicine, Shanxi Agricultural University, Taigu, China; ^2^Department of Veterinary Pharmacology and Toxicology, College of Veterinary Medicine, China Agricultural University, Beijing, China; ^3^Biomedical Sciences, SMART Pharmacology at Iowa State University College of Veterinary Medicine, Ames, IA, United States; ^4^Beijing Orbiepharm Co. Ltd., Beijing, China; ^5^College of Animal Science and Technology, Anhui Agricultural University, Hefei, China

**Keywords:** pharmacokinetics, vitacoxib, NSAIDs, cyclo-oxygenase inhibitors, COX-2

## Abstract

The objectives of this study were to elucidate absorption, tissue distribution, excretion, and metabolism of vitacoxib, a novel selective cyclooxygenase-2 inhibitor, in Wistar rats. Vitacoxib was detected in most tissues within 15 min, suggesting that it was well distributed. Moreover, it could cross the intestinal barrier. Vitacoxib was mainly eliminated as two metabolites. Nine proposed metabolites of vitacoxib were found in the plasma, bile, urine, and feces of rats. Two main metabolites, 4-(4-chloro-1-(5-(methyl-sulfonyl) pyridin-2-yl)-1H-imidazol-5-yl) phenyl methanol (M1) and 4-(4-chloro-1-(5-(methyl-sulfonyl) pyridin-2-yl)-1H-imidazol-5-yl) benzoic acid (M2), were identified in rat feces and urine. Further, the authentic standards of M1 and M2 were synthesized to confirm their structures. The carboxylic acid derivative was the major metabolite of vitacoxib excreted in the urine and feces. Hydroxylation of the aromatic methyl group of vitacoxib and additional oxidation of the hydroxymethyl metabolite to a carboxylic acid metabolite were the proposed metabolic pathways. Vitacoxib displayed a high *AUC*_*last*_ (4895.73 ± 604.34 ng·h/ml), long half-life (4.25 ± 0.30 h), slow absorption (*T*_*max*_, 5.00 ± 2.00 h), and wide tissue distribution in rats. Our findings provide significant information for the further development and investigation of vitacoxib as an effective nonsteroidal anti-inflammatory agent, and highly its potential for use future in a clinical setting.

## Introduction

Nonsteroidal anti-inflammatory drugs (NSAIDs) inhibit the activity of an important mediator during inflammation *in vivo*, namely the cyclo-oxygenase (COX) enzyme. There are at least two major isoforms of COX enzymes: COX-1 is responsible for the normal day-to-day “housekeeping” of biological activities, whereas COX-2 is primarily expressed to play a role during inflammation. Selective COX-2-inhibitors serve as a valuable therapeutic option in reducing pain and inflammation, and cause adverse gastrointestinal effects compared with nonselective NSAIDs due to their COX-1–sparing effect (1). Therefore, it was hypothesized that if an NSAID was developed that selectively inhibited COX-2, it might retain the desired anti-inflammatory property and lose the tissue-damaging effects associated with nonselective NSAIDs (2). However, clinical trials have revealed that COX-2 inhibitors are associated with an increased risk of cardiovascular events, with some drugs in this class having worse risks than the others (3). Therefore, rofecoxib by Merck and lumiracoxib have been withdrawn from the market (4) in several countries (3).

**Figure 1:**
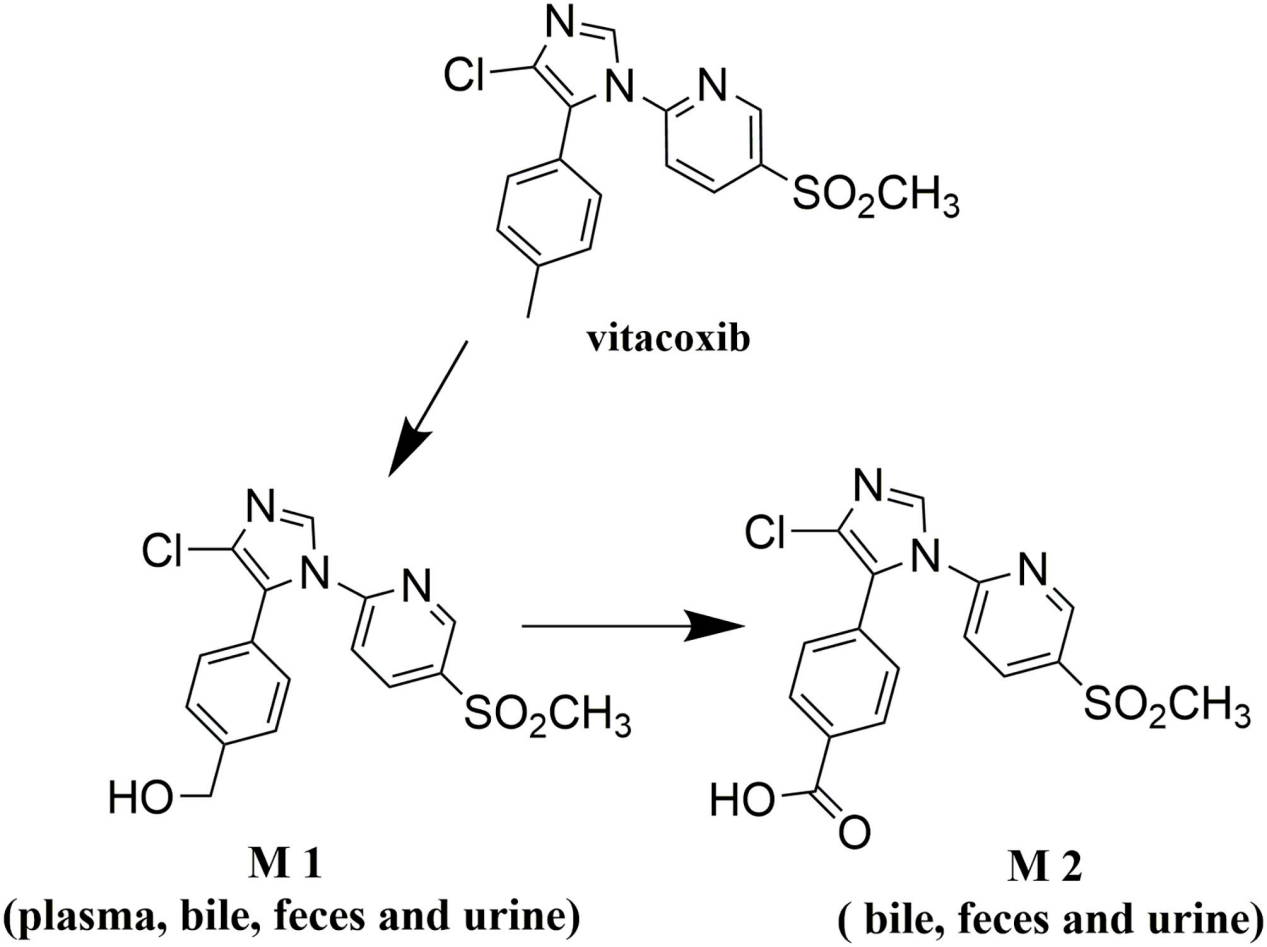
The proposed metabolic pathway for vitacoxib in rats.

Vitacoxib [2-(4-chloro-5-p-tolyl-1H-imidazol-1-yl)-5-(methyl sulfonyl) pyridine (C_16_H_14_ClN_3_O_2_S); Figure 1], a potent COX-2 inhibitor was developed and registered as a novel anti-inflammatory drug for veterinary use in China (5). Preclinical trials have shown that it specifically inhibits COX-2 in dogs and rats, and is likely to be a more viable candidate than celecoxib (6). To date, vitacoxib has solely been approved for use in dogs in China and has not been reviewed by the United States Food and Drug Administration (US FDA) or the European Medicines Agency.

Several toxicological studies have reported the oral LD_50_ of vitacoxib to be > 5,000 mg/kg body weight (BW) in Sprague Dawley (SD) rats and Institute of Cancer Research (ICR) mice, and the no-observed-adverse-effect-level (90 days and 180 days) was well tolerated up to a dose of 20 mg/kg and 6 mg/kg BW/day in SD rats after repeated dietary administration, respectively (3, 7). No mutagenicity or teratogenicity has been observed in rats and mice (8). A recent study has reported that vitacoxib does not cause skin irritation in rabbits or skin sensitization in guinea pigs (9). A few other studies have reported vitacoxib pharmacokinetics in dogs (10, 11), horses (12), cats (13, 14) and rabbits (15). It is well known that pharmacokinetics and tissue-distribution studies are paramount in drug development (16), during which a researcher can prognosticate various issues/target organs related to drug efficacy and toxicity (17). Moreover, absorption and metabolism are primary factors affecting drug bioavailability in animals after oral administration. The findings reported here are part of the development program to evaluate the pharmacokinetic parameters, tissue distribution, metabolism, and excretion of vitacoxib in rats after its oral administration.

## Materials and Methods

### Chemicals

Vitacoxib chewable tablets (30 mg/tablet) and pure vitacoxib powder (purity ≥ 99.8%, HPLC grade) were donated by Beijing Orbiepharm Co., Ltd (Beijing, PR China). Two main metabolites 4-(4-chloro-1-(5-(methyl sulfonyl) pyridin-2-yl)-1H-imidazol-5-yl) phenyl) methanol (M1) and 4-(4-chloro-1-(5-(methyl sulfonyl) pyridin-2-yl)-1H-imidazol-5-yl) benzoic acid (M2) (purity ≥ 98%) were synthesized by Beijing Orbiepharm Co., Ltd. Acetonitrile (HPLC grade), methyl tert-butyl ether (HPLC grade), and formic acid (LC/MS grade) were obtained from Fisher Scientific Co. (NJ, USA). Water for HPLC was purified using a Milli-Q Synthesis system (Millipore, MA, USA). Cyclophosphamide and carboxymethyl cellulose sodium (CMC-Na) were obtained from Tianjin Chemical Reagent Company (Tianjin, China). All other reagents and chemicals were of chromatography grade and supplied by Beijing Chemical Reagent Co., (Beijing, China).

### Animals

Male and female Wistar rats weighing 240–340 g were supplied by Beijing Vital River Laboratories (Charles River Laboratories). All animals were provided access to food and water *ad libitum* and allowed to acclimatize to a minimum of 2 weeks before initiating experiments. The major metabolites of vitacoxib were determined according to the US FDA guidelines, “Safety testing of drug metabolites (2008)”. Experiments involving animal use were reviewed and approved by the Institutional Animal Care and Use Committee of the China Agricultural University prior to commencing the study (WTPJ20160003).

### Pharmacokinetics Studies

Five male rats were administered a single dose of vitacoxib (18 mg/kg) obtained by crushing and dissolving vitacoxib tablets in 0.5% CMC-Na (6 mg/ml). Blood samples (0.3 ml) were collected via a cannula directly into heparinized tubes before drug administration and 5, 15, 30 min, and 1, 2, 3, 4, 6, 8, 10, 12, 24, 36, and 48 h after drug administration. The samples were centrifuged at 2,280 × *g* for 10 min as soon as possible and stored at −20°C until further analysis (within 21 days).

Vitacoxib in plasma samples was analyzed using an established and validated UPLC-MS/MS (Waters Acquity UPLC and Water Quattro Premier, Waters Co., USA) method using a procedure previously described by our group (18). The lower limit of quantification was 0.5 ng/ml and the calibration range of the method was 0.5–1,000 ng/ml. Noncompartmental analysis was performed to determine the pharmacokinetic parameters, as appropriate, using WinNonlin^TM^ (version 6.4, Certara, Princeton NJ, USA) computational software.

### Tissue-Distribution Studies

Eighteen rats were administered a single oral dose (18 mg/kg BW) of vitacoxib (tablets crushed and dissolved in 0.5% CMC-Na, 6 mg/ml). Six rats were euthanized at each time point of 0.25, 6, and 24 h after dose administration. The brain, liver, heart, spleen, lungs, bladder, kidneys, gracilis muscle, gastrointestinal tract, large intestinal contents, small intestine, bone marrow, testes, epididymis, thymus gland, fat, and pancreas were collected and weighed. Each sample was diluted with 3 volumes (v/w) of triple-distilled water and homogenized, and the homogenate was stored at −20°C until further analysis. Vitacoxib in tissue samples was analyzed using UPLC-MS/MS by following a previously described procedure.

### Urinary, Fecal Excretion, and Bile Excretion

Six male rats were placed in separate metabolic cages and administered a single oral dose of 18 mg/kg of vitacoxib (tablets were crushed and dissolved in 0.5% CMC-Na, 6 mg/ml). Urine and fecal samples were collected from each animal in metabolic cages at different time points at 2, 4, 6, 8, 10, 14, 24, 36, 48, 60, 72, 84, 96, 108, 120, 132, 144, 156, and 168 h after drug administration. The total weight of feces and urine was recorded after collection.

Eight male rats with bile-duct cannulation were administered 18 mg/kg of vitacoxib (tablets crushed and dissolved in 0.5% CMC-Na, 6 mg/ml). Rats were housed in metabolism cages and bile samples were collected at 2, 4, 6, 8, 10, 14, 24, 36, 48, 60, and 72 h after drug administration. All samples were stored at −20°C until further analysis. Stability studies were conducted to ensure that vitacoxib was stable in the feces and urine during the excretion study (data not shown). Vitacoxib in urine, fecal, and bile samples was determined via validation UPLC-MS/MS using the procedure described for blood (18).

### Metabolism in Plasma, Urine, and Bile Samples

Six male rats were placed in their own metabolic cages and orally administered a single dose of 18 mg/kg dose of vitacoxib (tablets crushed and dissolved in 0.5% CMC-Na, 6 mg/ml). Bile, fecal, and urine samples were collected from each animal in metabolic cages at 0~8, 8~23, 23~32, 32~48, 48~58, 58~72, 72~82, 82~96, and 96~128 h after administration. The total weight of urine and feces was recorded after collection. Feces samples were diluted with 3 volumes (v/w) of triple-distilled water and homogenized, and the homogenate was stored at −20°C until further analysis.

Chromatographic separation was performed using an ACQUITY UPLC^TM^ system (Waters Corporation, Milford, MA, USA). A Waters ACQUITY UPLC HSS T3 column (1.7 μm, 2.1 mm × 100 mm) was used for reversed-phase UPLC and the column temperature was maintained at 40°C. The flow rate of the mobile phase was 0.5 ml/min and the injection volume was 5 μl. Mobile phase A was 0.1% formic acid in water and mobile phase B was 0.1% formic acid in methanol. The column was eluted using a linear gradient (0 → 1 min, phase A 90%; 1 → 10 min, phase A 90% → 5%; 10 → 13.5 min, phase A 5% → 5%; and 13.5 → 15 min, phase A 5% → 90%).

MS analysis was performed using a Xevo G2-S Q-TOF mass spectrometer. The eluent was not shunted but injected directly into the mass analysis system in the positive-ion mode. The source temperature was set at 120°C. The flow rate of the desolvation gas was 800 L/h at a temperature of 500°C. The capillary voltage was set at 3 kV with 35 V as the cone voltage. Methods to analyze different tissues and samples were developed and validated in our lab. Data were analyzed using Metabolynx^TM^ software (version 4.1), which could automatically identify metabolites by comparing treatment samples with the respective controls.

## Results

### Pharmacokinetics

**Figure 2:**
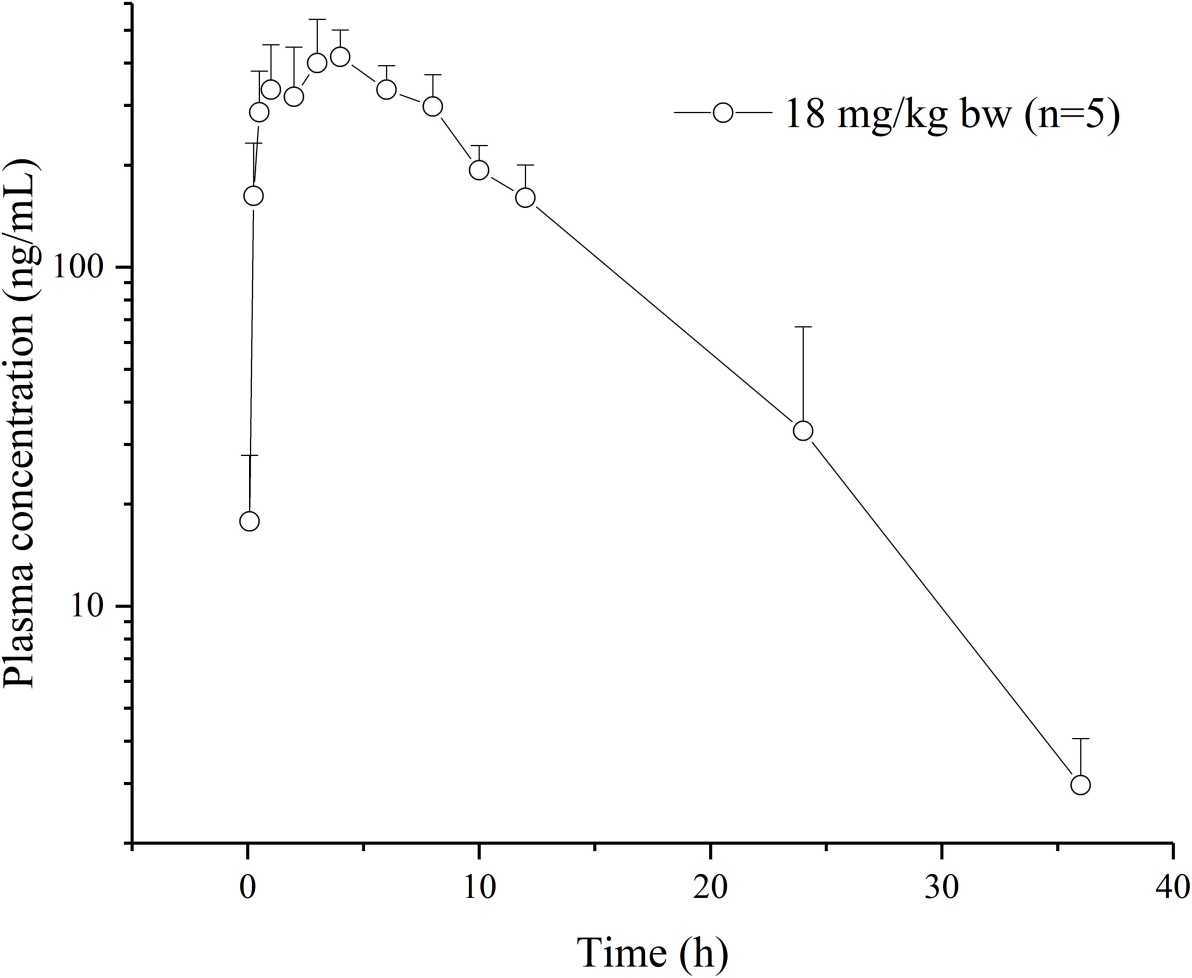
Plasma concentration-time curves of vitacoxib in rats (*n* = 10) after an oral administration of 18 mg/kg bw.

No obvious or serious adverse effects were exhibited by rats after a single oral dose of the drug (18 mg/kg BW). Plasma concentration–time curves are plotted on a semilogarithmic scale (Figure 2). The relevant pharmacokinetic parameters are listed in Table 1.

**Table 1:** Plasma pharmacokinetic parameters after a single p.o. administration of 18 mg/kg b.w. vitacoxib.

**Parameter (units)**	**Route of administration**
	**p.o. 18 mg/kg (*n* = 5)**
*λz* (1/h)	0.16 ± 0.01
*T_1/2λ*z*_* (h)	4.25 ± 0.30
*T_*max*_* (h)	5.00 ± 2.00
*C_*max*_* (ng·ml^−1^)	450.19 ± 96.23
*AUC_*last*_* (ng·h·ml^−1^)	4895.73 ± 604.34
*AUC_*INF*_*obs*_* (ng·h·ml^−1^)	4934.65 ± 588.37
*MRT_*last*_* (h)	8.09 ± 1.72

*Pharmacokinetic parameters were calculated using Non-Compartmental Analysis Model 200 in WinNonlin™ software (version 6.4; Certara USA); λz, the elimination rate constant; T_1/2λz_, elimination half-life; T_max_, the time after initial injection to when C_max_ occurs; C_max_, maximum plasma concentration; AUC_last_, area under the concentration vs. time curve from 0 to last point; AUC_INF_obs_, area under the concentration versus time curve from 0 to infinity; MRT_last_, mean residence time*.

### Tissue Distribution

**Figure 3:**
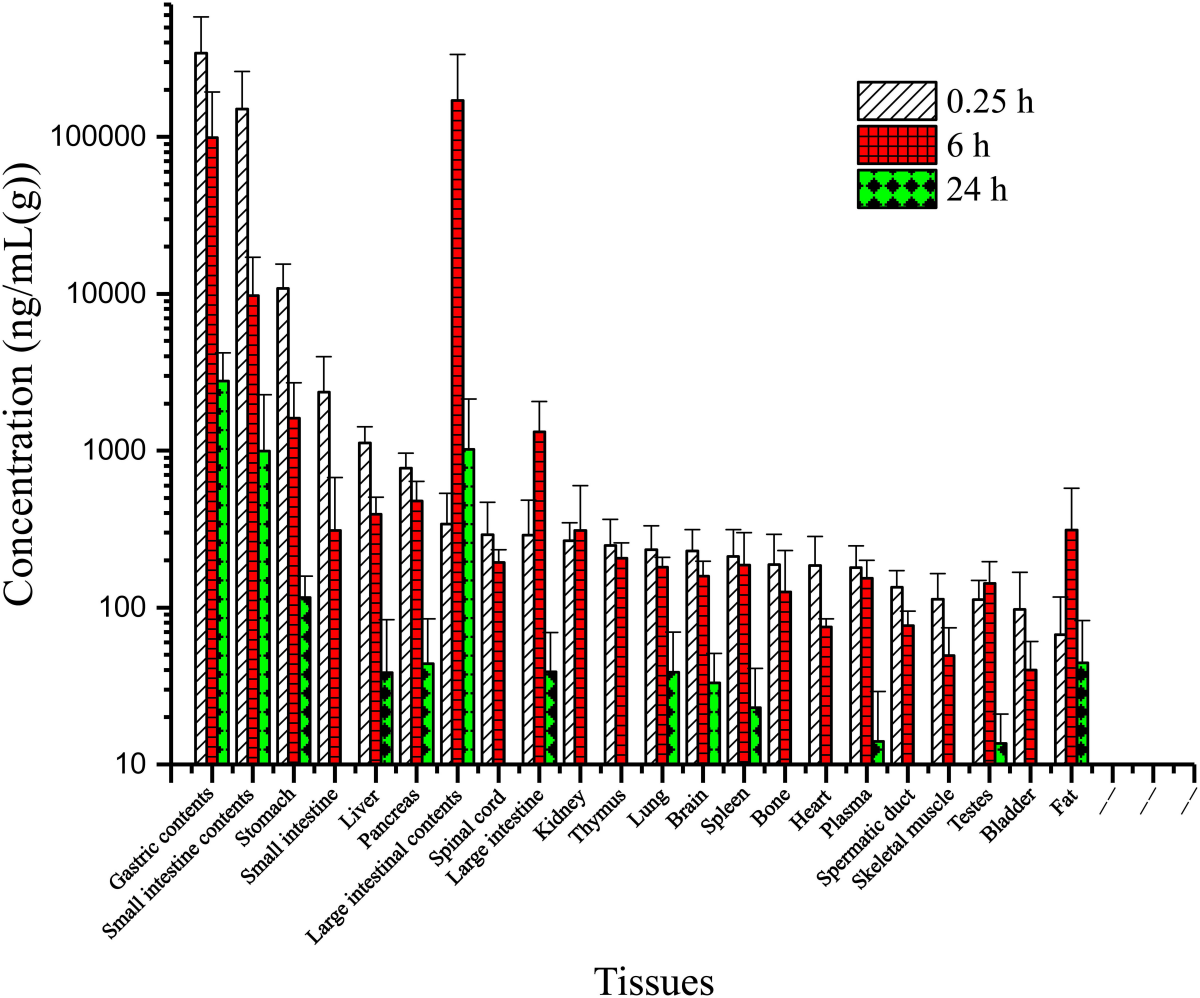
Tissue distribution of vitacoxib in rats after a single oral gavage dose of 18 mg/kg bw.

**Figure 4:**
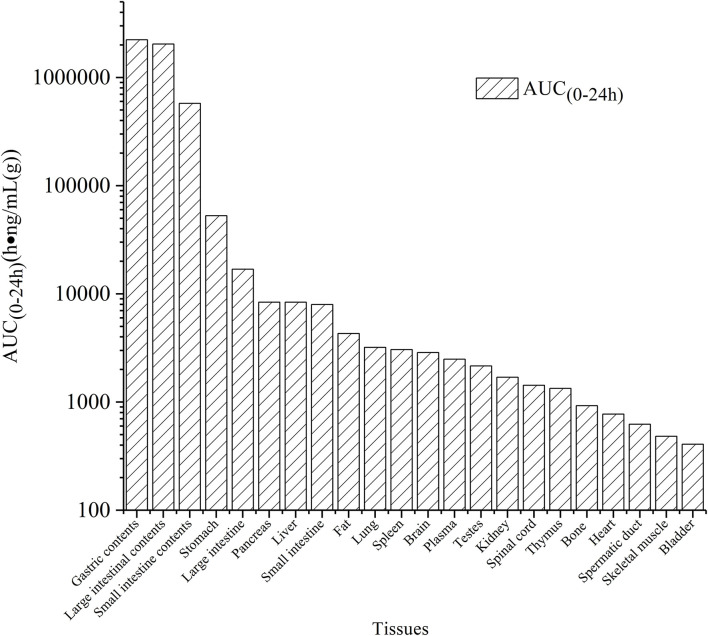
AUC_(0~24*h*)_ values of vitacoxib in different tssue after a single oral gavage dose of 18 mg/kg bw.

The tissue distribution of vitacoxib in various tissues was characterized after a single oral dose (18 mg/kg BW). The tissue concentration vs. time profiles are depicted in Figure 3 and the area under the curve *AUC*_(0~24*h*)_ values in different tissue are shown in Figure 4. Vitacoxib was seen in the majority of tissues by 15 min, suggesting that it was well distributed in tissues and also extensively distributed throughout the body in rats (Figure 3).

### Urinary, Fecal Excretion, and Bile Excretion

**Figure 5:**
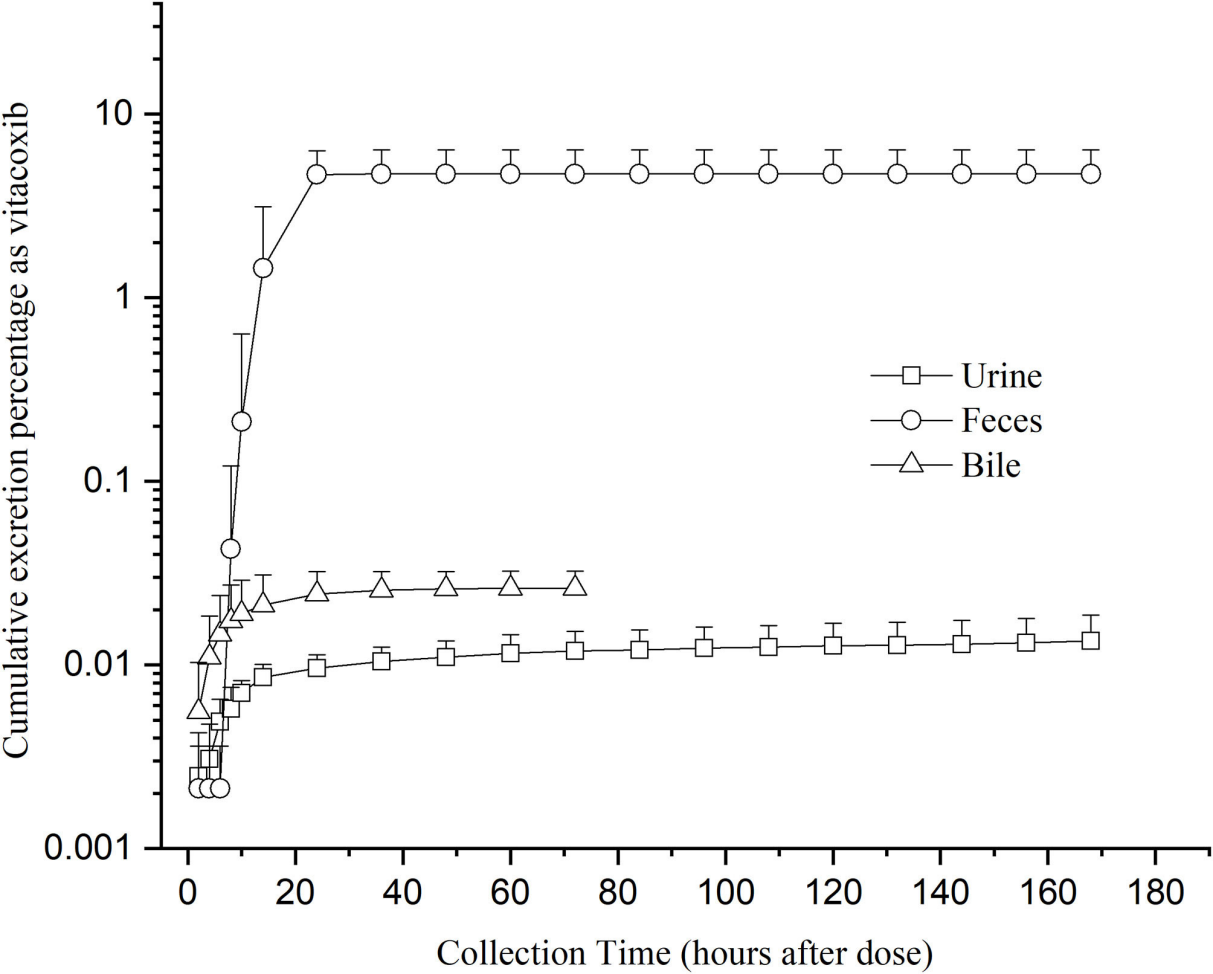
Mean cumulative excretion percentage as vitacoxib after a single oral gavage dose of 18 mg/kg bw.

Unchanged vitacoxib was found in the bile, feces, and urine. The cumulative excretion percentage as vitacoxib after a single oral dose is given in Figure 5. After a single oral dose of vitacoxib, 0.02610% ± 0.00627% (0~72 h), 0.0135% ± 0.0053% (0~168 h) and 4.7334% ± 1.6645% (0~168 h) was recovered in the bile, urine and feces, respectively, in its unaltered form.

### Metabolites of Vitacoxib Present in Plasma, Urine, and Feces

**Figure 6:**
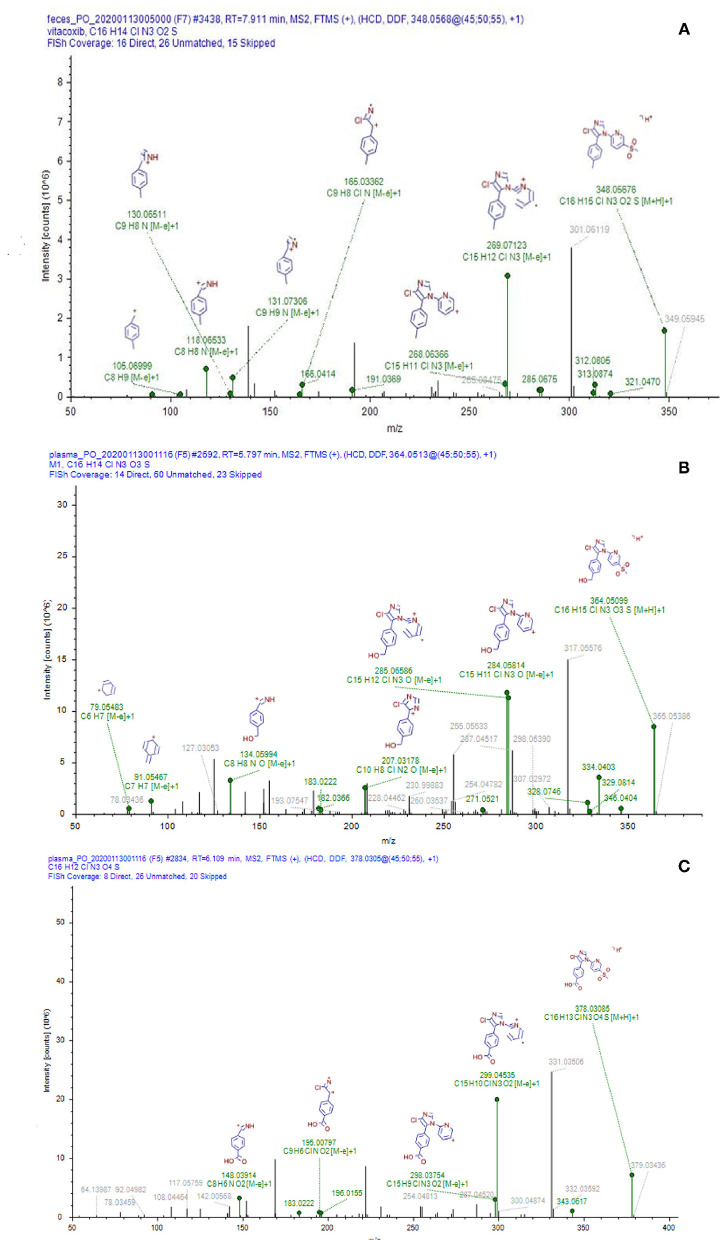
Representative daughter-ion mass spectrum and chemical structure of vitacoxib **(A)**, M1 **(B)** and M2 **(C)**.

**Figure 7:**
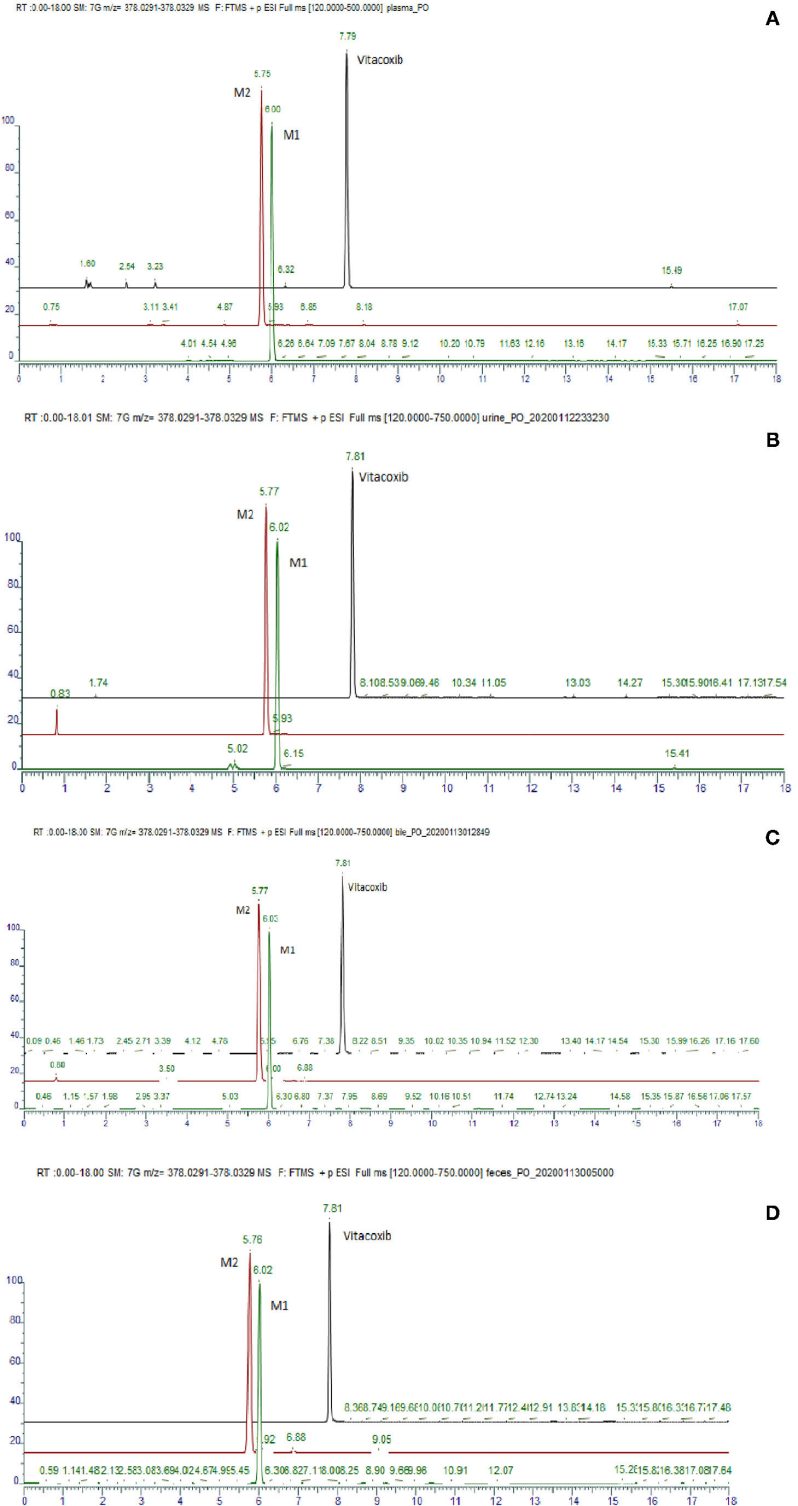
Typical ion chromatogram obtained from the analysis of the pool of plasma **(A)**, urine **(B)**, bile **(C)** and feces **(D)**.

Nine proposed metabolites of vitacoxib in rats were detected using Xevo G2-S Q-TOF (Table 2, Table S1). Several minor metabolites were identified in the feces extract and urine (Figure SA). Notably, seven urinary metabolites were identified. The majority of urinary and fecal metabolites consisted of the carboxylic acid derivatives of vitacoxib (Figure SA). In this study, two metabolites from the plasma/bile/feces/urine extracts were identified using MS and designated M1 and M2 (Figures 6, 7). Authentic standards of M1 and M2 were synthesized to further confirm their structures. The chemical structures of M1 and M2 were identified as 4-(4-chloro-1-(5-(methyl sulfonyl) pyridin-2-yl)-1H-imidazol-5-yl) phenyl) methanol and 4-(4-chloro-1-(5-(methyl sulfonyl) pyridin-2-yl)-1H-imidazol-5-yl) benzoic acid, respectively.

**Table 2:** Structures of metabolites (M1 and M2) of vitacoxib in rats.

**Metabolite**	**[M+H]+**	**Proposed structure**	**Source**
M1	364	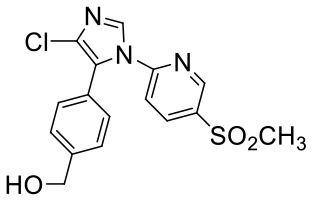	Plasma, bile, feces, and urine
M2	378	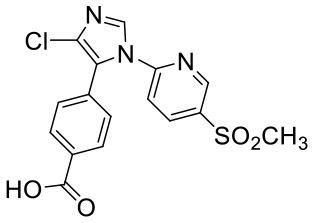	Bile, feces, and urine

## Discussion

In this study, we investigated the pharmacokinetics, distribution, metabolism, and excretion of vitacoxib, a novel COX-2 inhibitor, in rats.

The dose of vitacoxib administered to rats in our pharmacokinetics study was higher than that approved for dogs in clinical practice (2 mg/kg BW). However, no adverse effects of the drug were noted in rats during the study. Vitacoxib was absorbed slowly from the gastrointestinal tract and a maximum concentration of 450.19 ± 96.23 ng/ml was observed at around 5.00 ± 2.00 h following oral administration. Some studies have reported the pharmacokinetics of vitacoxib in dogs (10), horses (12), cats (13) and rabbits (15). The average *T*_1/2λ*z*_ (h) of vitacoxib in rats was shorter than that reported in earlier studies in horses (*T*__1/2λ*z*__, 7.39 ± 4.17 h, 0.1 mg/kg bw) and dogs (*T*__1/2λ*z*__, 5. 87 ± 3. 37 h, 2 mg/kg bw). The *C*_*max*_ (ng/ml) and *AUC*_*last*_ (μg·h/ml) of vitacoxib in rats were higher than those in horses (12) and dogs (11).

Tissue-distribution findings suggested that vitacoxib could cross the intestinal barrier. The *AUC*_(0~24*h*)_ values in different tissues were in the order of gastric contents > large intestinal contents > small intestinal contents > stomach > large intestine > pancreas > liver > small intestine > fat > lungs > spleen > brain > plasma > testes > kidneys > spinal cord > thymus > bones > heart > spermatic > skeletal muscles > bladder (Figure 4). In light of these characteristics, it was particularly interesting to observe that vitacoxib was found in the brain, implying that it could easily cross the blood-brain barrier. Moreover, its distribution in the gastrointestinal tract, liver, kidneys, heart, and lungs was higher than that in the other organs that were examined (Figure 3). By 24 h after vitacoxib administration, its concentration in most tissues was below the limit of detection, indicating that there was no drug retention in the experimental animals.

After the single oral dose used to determine drug elimination, vitacoxib was found to be extensively and widely metabolized before excretion, which was < 5% of the dose recovered in the urine or feces as the parent drug. The low concentration of drug in the urine was one of the main findings that suggested that vitacoxib was metabolized by cytochrome P-450 (CYP) enzymes in the liver and that vitacoxib was likely to be excreted as metabolites. These results indicated that vitacoxib was eliminated from the kidneys into the urine and that the liver was the main site for vitacoxib metabolism. Similarly, low amounts of the drug in feces might be because the drug was mainly retained in intestinal tissues, where it could also be metabolized by CYP. Vitacoxib was eliminated mainly as its metabolites. Our findings suggested that vitacoxib was mainly excreted in the feces and urine. However, further research is necessary to obtain more detailed information with respect to the quantitative determination of vitacoxib and its metabolites in plasma and excreta.

Nine proposed metabolites of vitacoxib in rats were detected in metabolic experiments. Our findings suggested that the absorbed vitacoxib underwent extensive metabolism and was further biotransformed into numerous minor metabolites in rats, which were excreted in the urine and feces. Identification studies on the two metabolites revealed that the methyl group of vitacoxib was initially oxidized to a hydroxymethyl metabolite, followed by further oxidation to a carboxylic metabolite (Figure 1). It should be noted that all minor metabolites and/or undiscovered metabolites were not quantified in this study. Thus, further investigations are needed to better understand and clarify the complete fate of vitacoxib. The carboxylic acid metabolite was the primary metabolite of vitacoxib excreted in the urine and feces. A minor amount of the dose was excreted as the hydroxymethyl metabolite in the feces and urine of rats.

## Conclusions

This is the first study to report the pharmacokinetics of vitacoxib in rats. Vitacoxib was found to be readily distributed in most tissues and to cross the blood-brain barrier in rats. It was extensively metabolized, and minimal unchanged drug was detected in the urine and feces of the experimental animals. Hydroxylation of the aromatic methyl group of vitacoxib and additional oxidation of the hydroxymethyl metabolite to a carboxylic acid metabolite is the proposed metabolic pathway of vitacoxib. These results furnish significant information for the further development and investigation of vitacoxib as an effective NSAID and for its potential use in a clinical setting. Moreover, results of drug distribution in tissues based on our *in vivo* studies will be useful in the development of pharmacokinetic/pharmacodynamic models to simulate animal and human studies.

## Data Availability Statement

The original contributions presented in the study are included in the article/Supplementary Material, further inquiries can be directed to the corresponding authors.

## Ethics Statement

The animal study was reviewed and approved by the Institutional Animal Care and Use Committee of the China Agricultural University.

## Author Contributions

XC, FS, and JW contribute to study design, execution, and give final approval of the manuscript. JK, JQ, LZ, and XG contributed to animals experiments and data analysis. JL donated the test drugs. JW were involved in study execution, data analysis and interpretation, and manuscript preparation. All authors contributed to the article and approved the submitted version.

## Funding

This work was supported by Central Funds Guiding the Local Science and Technology Development in Shanxi Province (YDZJSX2021A034); Fund Program for the Scientific Activities of Selected Returned Overseas Professionals in Shanxi Province (20210012); Project of Scientific Research for Excellent Doctors, Shanxi Province, China (SXBYKY2021047); Research Fund (Clinical Diagnosis and Treatment of Pet) for Young College Teachers in Ruipeng Commonweal Foundation (RPJJ2020021). Project of Science and Technology Innovation Fund of Shanxi Agricultural University (2021BQ06).

## Conflict of Interest

JL is employed by Beijing Orbiepharm Co. Ltd. The remaining authors declare that the research was conducted in the absence of any commercial or financial relationships that could be construed as a potential conflict of interest.

## Publisher's Note

All claims expressed in this article are solely those of the authors and do not necessarily represent those of their affiliated organizations, or those of the publisher, the editors and the reviewers. Any product that may be evaluated in this article, or claim that may be made by its manufacturer, is not guaranteed or endorsed by the publisher.
