# Supplementary material for: Pharmacokinetics, Tissue Distribution, Metabolism and Excretion of a Novel COX-2 Inhibitor, Vitacoxib, in Rats
Source: Front Vet Sci. 2022 Apr 8;9:884357. doi: 10.3389/fvets.2022.884357 (PMC9024361; doi:10.3389/fvets.2022.884357)
Supplement: Supplementary file 1 [file Table_1.DOCX]

Supplementary Materials

Supplementary _Table 1. Structures of Metabolites (M3 to M9) of vitacoxib in rats

| **Metabolite** | [M+H]+ | Proposed Structure | Source |
| --- | --- | --- | --- |
| M3 | 188 |  | Plasma |
| M4 | 158 |  | Plasma and bile |
| M5 | 194 |  | Bile |
| M6 | 330 |  | Urine |
| M7 | 360 |  | Urine |
| M8 | 344 |  | Feces and urine |
| M9 | 223 |  | Urine |


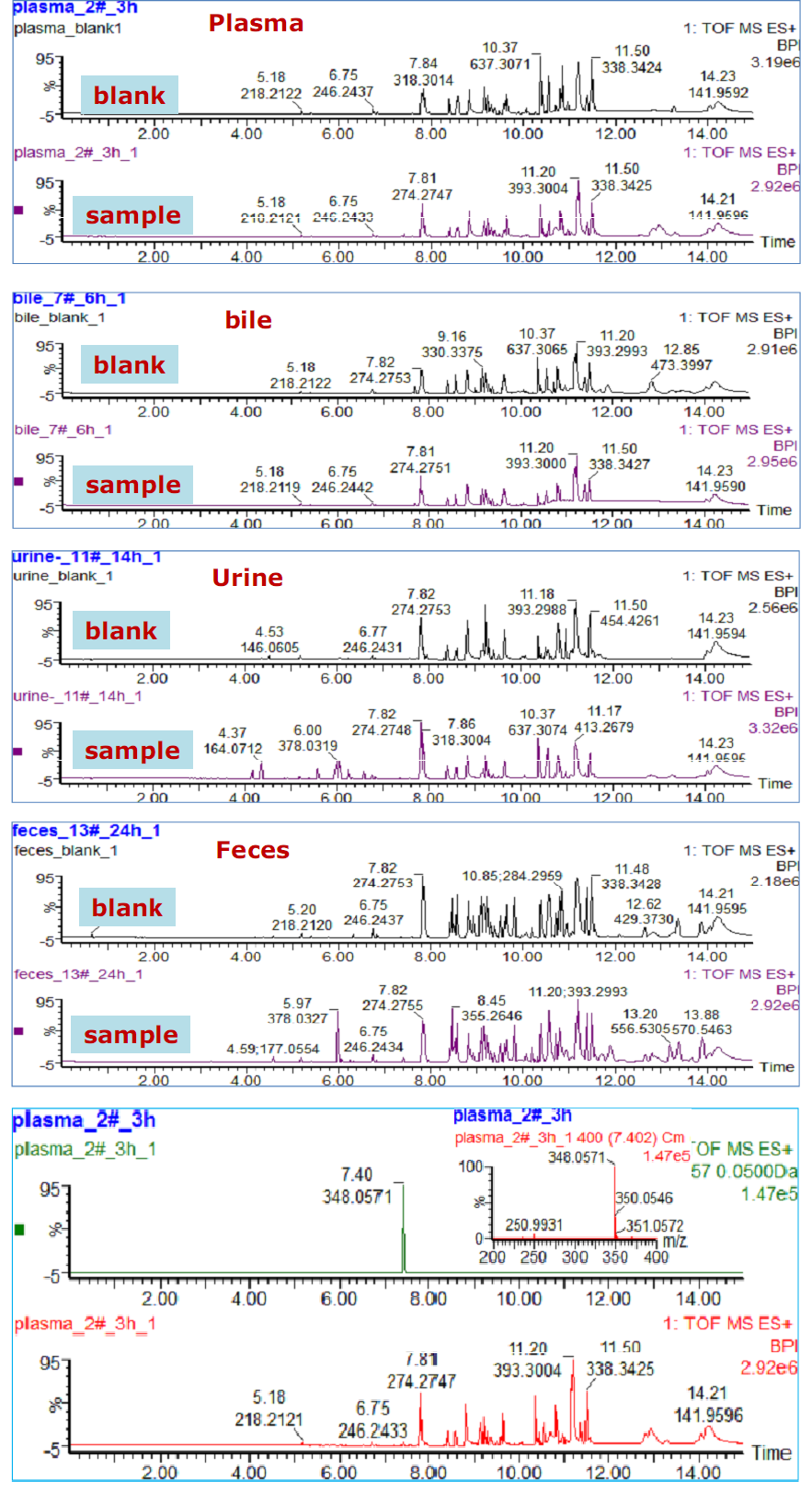


**Supplemental Figure (A).** Several minor metabolites were identified in the plasma/bile/feces/urine extracts.
